# Work engagement and occupational future time perspective among Chinese new nurses: the mediating role of career adaptability

**DOI:** 10.3389/fpsyg.2025.1676821

**Published:** 2026-01-09

**Authors:** Ying Shen, Fengfeng Song, Aini Lv, Yuanyuan Su, Le Lu, Yan Song

**Affiliations:** Department of Rehabilitation, Tangdu Hospital, Fourth Military Medical University, Xian, China

**Keywords:** work engagement, occupational future time perspective, career adaptability, new nurses, nursing workforce

## Abstract

**Introduction:**

The aim of this study is to investigate the relationship between work engagement, occupational future time perspective (OFTP), and career adaptability among newly graduated nurses (NGNs) in China.

**Methods:**

A questionnaire survey was conducted with 316 newly graduated nurses from 20 hospitals across various provinces in China. The survey included measures of work engagement, OFTP, and career adaptability. Structural equation modeling was used to analyze the data and test the mediating role of career adaptability.

**Results:**

In this study, the mean scores of work engagement, occupational future time perspective and career adaptability among 316 newly graduated were (34.55 ± 10.61), (28.56 ± 8.16), and (68.31 ± 15.77), respectively. The findings indicate that a positive OFTP significantly contributes to higher levels of work engagement among NGNs. Career adaptability was found to mediate the relationship between OFTP and work engagement. Married nurses, those with formal employment contracts, and those with more experience or fewer night shifts reported higher levels of work engagement.

**Conclusion:**

This study underscores the importance of a positive OFTP in enhancing work engagement among NGNs. Career adaptability plays a crucial mediating role, suggesting that interventions aimed at improving adaptability could amplify the positive effects of a positive OFTP on work engagement.

## 1 Introduction

In recent years, the nursing profession has experienced substantial changes, especially in China, where the healthcare system confronts increasing demands from an aging population and a rise in chronic diseases. Currently, a global shortage of nurses is intensifying, with the situation in China worsening significantly (Drennan and Ross, 2019). The COVID-19 pandemic has exacerbated this shortage, highlighting the urgent need for additional newly graduated nurses (NGNs) to engage in clinical work (NCSBN, 2024). The Royal College of Nursing defines NGNs as nurses with 0–24 months of clinical experience or within 3 years post-graduation (Murray et al., 2018). These new nurses frequently face challenges in work engagement due to the demanding nature of the role, inexperience, and the transition to new work environments (Frögéli et al., 2019). Work engagement—characterized by vigor, dedication, and absorption—is essential in nursing due to its direct effects on patient care quality, job performance, and retention rates (Alharbi et al., 2019; Moloney et al., 2018; Van Bogaert et al., 2014). Elevated levels of engagement correlate with improved health outcomes, enhanced patient satisfaction, and lower nurse turnover (Halm, 2019; Moloney et al., 2018). Studies indicate that, compared to developed countries, the work input from nurses in China is markedly low, suggesting significant potential for enhancement (Ye et al., 2020). Furthermore, the engagement levels among NGNs are notably lower than those of more experienced nurses (Fan et al., 2024; Sun et al., 2022).

The primary factors influencing nurses' job involvement include individual, organizational, and family aspects (Lambert et al., 2017; Lin et al., 2024). Time, however, is a crucial but under explored factor impacting human behavior and nurses' job involvement. The concept of occupational future time perspective (OFTP) emerges from Carstensen's definition of future time perspective as a flexible cognitive-motivational structure shaped by an individual's perception of their remaining life opportunities, which evolves with life experiences (Carstensen and Lang, 2022). Zacher and Frese expanded this concept, applying it to the workplace to formulate OFTP, a significant element in career development (Zacher and Frese, 2009). OFTP encompasses an individual's perception of their professional future, including goals, aspirations, and opportunities (Rudolph and Zacher, 2018). A positive OFTP can drive employees to invest in their career development and sustain high work engagement (Kooij and Van De Voorde, 2005). For novice nurses, a clear and optimistic OFTP is vital for overcoming initial career hurdles.

Proposed by Savickas (1997), the concept of career adaptability encompasses the skills and competencies necessary for career development, particularly in adapting to unexpected changes. Additionally, the career adaptability model comprises four components: concern, control, curiosity, and confidence, each addressing different aspects of career planning and preparedness (Hirschi and Valero, 2015). Notably, adaptability influences individual positive attributes, thereby impacting work engagement (Yang et al., 2019).

Although the significance of work engagement and occupational future time perspective in nursing is well-acknowledged, the relationships between OFTP, career adaptability, and work involvement among new nurses are not well understood. Furthermore, the potential mediating role of career adaptability between work engagement and OFTP warrants deeper investigation. This study aims to elucidate these relationships, to offer insights on how to support new nurses in improving their work engagement and career prospects.

## 2 Literature overview

### 2.1 Occupational future time perspective and work engagement

Occupational future time perspective (OFTP) refers to how individuals perceive their future in their occupational roles (Henry et al., 2017). It encompasses career goals, aspirations, and perceived opportunities. OFTP plays a crucial role in career planning and development. A positive OFTP can motivate individuals to invest in their career development, seek new opportunities, and remain committed to their professional roles (Day and Allen, 2004). Conversely, a negative OFTP may lead to disengagement, reduced job satisfaction, and higher turnover intentions (Lorente et al., 2014). For new nurses, having a clear and positive OFTP can help them navigate the initial challenges of their careers, set long-term career goals, and remain motivated despite the demanding nature of their work (Baharum et al., 2023).

Kahn proposed in 2019 that work engagement is the process of adaptation between individual role and job role (Kahn, 2019). When the personal role is well adapted to the work role, individuals will express themselves through physiological, cognitive, and emotional means, which not only ensures successful job completion but also helps establish good interpersonal relationships (Mohanty and Sussman, 2013). With the continuous improvement of work involvement theory and growing scholarly attention, research on nurses' work involvement has expanded internationally. Cao and Lu (2021) believe that nurses who hold a positive view of their career future are not only enthusiastic about their work but also willing to contribute to their organization and reluctant to leave their current job. Therefore, we hypothesize:

H1: Occupational future time perspective is positively and directly related to new nurses' work engagement.

### 2.2 Career adaptability as a mediator

Career adaptability is defined as the readiness and resources to cope with career-related changes and challenges (Savickas, 2005). Savickas conceptualized career adaptability as a multi-dimensional construct comprising concern, control, curiosity, and confidence (Savickas, 1997). (1) Career concern involves planning and preparing for the future, considering career possibilities, and developing a sense of direction. (2) Career control refers to taking responsibility for one's career, making decisions, and exerting influence over one's career path. (3) Career curiosity entails exploring possible selves and future scenarios, being open to new experiences, and seeking information. (4) Career confidence involves believing in one's ability to achieve career goals and overcome obstacles.

Research has demonstrated that occupational future time perspective (OFTP) is linked to various job-related outcomes, including job performance, satisfaction, and turnover intentions (Medina, 2013). For instance, Zacher and Rudolph (2021) found that employees with a positive OFTP were more likely to engage in proactive career behaviors, such as seeking development opportunities and building professional networks. Furthermore, OFTP influences work engagement, with individuals perceiving more future opportunities reporting higher job satisfaction and lower burnout levels (Henry et al., 2017).

Career adaptability is crucial for successful career development, especially in dynamic and uncertain work environments (Hirschi, 2012). It allows individuals to navigate career transitions, adapt to new roles, and respond to labor market changes. For new nurses, career adaptability helps manage the stress and challenges associated with transitioning from education to professional practice (Baharum et al., 2023).

Several studies have examined the relationship between career adaptability, work engagement, and OFTP. Hirschi (2012) found that career adaptability positively influences work engagement by providing individuals with the resources to cope with career-related challenges. Additionally, a clear and positive perception of future time improves individuals' career adaptability (Li et al., 2025). Therefore, career adaptability acts as a key mediator linking work engagement and OFTP, especially for new nurses facing early career challenges. Taken together, we propose:

H2: Career adaptability mediates the relationship between occupational future time perspective and work engagement.

## 3 Methods

### 3.1 Study design

A cross-sectional design was used in this study.

### 3.2 Participants and data collection

From October 2023 to August 2024, a convenience sampling method was used to select newly registered nurses from 20 hospitals across Shaanxi, Shandong, Ningxia, Zhejiang, Jiangsu, Sichuan, Jiangxi, Xinjiang, Beijing, Yunnan, and Guangdong provinces were selected as the research subjects. The inclusion criteria were: (1) possession of the professional qualification certificate for nurses from the People's Republic of China, (2) less than three years of formal work experience, and (3) informed consent and voluntary participation. According to the sample size estimation method, the sample size should be at least five times the number of items. Thus, the required sample size *N* = (9 + 24 + 10) × 5 = 215.The required sample size for this study was estimated based on recommendations for structural equation modeling (SEM). For models of moderate complexity with three latent variables (Occupational Future Time Perspective, Career Adaptability, and Work Engagement) and multiple observed indicators per construct, methodological guidelines suggest a minimum of 200 participants to ensure model stability (Wolf et al., 2013). Ultimately, the study surveyed 316 newly registered nurses. All participants agreed to complete the questionnaire electronically using the Questionnaire Star platform.

### 3.3 Measures

#### 3.3.1 General demographic questionnaire

Gender, age, marital status, educational level, professional title, years of experience, department, night shift status, and the average number of night shifts per month were included in the general demographic questionnaire.

#### 3.3.2 Work engagement

The Utrecht Work Engagement Scale (UWES) was used to measure work engagement. The scale was developed by Schaufeli et al. in 2002 (Tong et al., 2020) and translated by Zhang and Gan (2005), to assess work engagement across different cultural backgrounds and occupational groups. The scale includes nine items across three dimensions: vitality, dedication, and concentration. A seven-point Likert scale was adopted. Each item is scored from one to seven points, ranging from “never” to “always”. A higher score signifies a greater level of work engagement. The Cronbach's alpha coefficient of the scale was 0.94. In this study, the Cronbach's alpha coefficient for this scale was 0.89.

#### 3.3.3 Occupational future time perspective

The Occupational Future Time Perspective Scale, developed by Zacher and de Lange in 2011, and validated in China by Wang et al. (2023). It is divided into two subscales: Open Career Future Time View and Limited Career Future Time View, comprising a total of ten items. Each item is rated on a five-point Likert scale, from one (strongly disagree) to five (strongly agree). The Cronbach's alpha coefficients for the two subscales are 0.83 and 0.85, respectively. In this study, the Cronbach's alpha coefficient for this scale was 0.92.

#### 3.3.4 Career adaptability

We used the Career Adapt-Abilities Scale (CAAS) compiled by Savickas and Porfeli (2012), which was translated and validated by Hou et al. (2012). The scale contains four dimensions: Career Concern (six items), Career Control (six items), Career Curiosity (six items), and Career Confidence (six items), totaling 24 items. A five-point Likert scale was used, with scores ranging from one (completely inconsistent) to five (completely consistent). The total score ranges from 24 to 120, with higher scores indicating greater career adaptability. The Cronbach's alpha coefficient for this scale is 0.89; in this study, it was 0.90.

### 3.4 Procedures for data collection

Data were collected through an online survey platform to ensure accessibility and convenience for participants. The survey link will be distributed via email and social media, accompanied by a brief introduction to the study and instructions for completing the survey. Participants will be assured of the confidentiality and anonymity of their responses.

### 3.5 Statistical analysis

SPSS 26.0 was used for data analysis, and AMOS 21.0 was utilized to construct the structural equation model. Categorical data were presented as frequencies and percentages. Continuous data that were normally distributed were expressed as mean ± standard deviation. Univariate ANOVA or independent sample *t*-tests were used to compare differences in nurses' work involvement, occupational future time perspective, and career adaptability across general demographic variables. Multiple linear regression was used to further analyze factors affecting work engagement. Pearson correlation analysis was conducted to examine the relationships between nurses' work engagement, occupational future time perspective (OFTP), and career adaptability. The structural equation model was employed to investigate the mediating role of career adaptability in the relationship between nurses' future time perspective and work engagement. The mediation hypothesis was tested using the Bootstrap method with 5,000 resamples, statistical significance of the indirect effects was determined when the 95% biascorrected confidence intervals did not include zero. The significance level was set at α = 0.05.

## 4 Results

### 4.1 Descriptive statistics

The study included a total of 316 new nurses from various healthcare settings in China. The demographic characteristics of the participants are summarized as follows: gender: 291 (92.1%) female and 25 (7.9%) male; the mean age of the participants was 26.53 ± 2.01 years. The majority of participants were single (61.1%), 79.7% had a bachelor's degree in nursing, and 28.8% of the participants had less than 1 year of work experience. The distribution of participants' demographics is shown in Table 1.

**Table 1:** Sociodemography information of participant nurses (*N* = 316).

**Characteristics**	***N* (%)**
**Gender**	
Female	291 (92.1%)
Male	25 (7.9%)
**Education**	
Postgraduate	52 (16.5%)
Undergraduate	252 (79.7%)
Junior college	12 (3.8%)
**Marital status**	
Single	193 (61.1%)
Married	123 (38.9%)
**Title**	
Nurse	140 (44.3%)
Nurse practitioner	108 (34.2%)
Supervisor nurse	68 (21.5%)
**Employment types**	
Temporary	263 (83.2%)
Permanent	53 (16.8%)
**Working years**	
< 1	91 (28.8%)
1–2	64 (20.3%)
>2–3	161 (50.9%)
**Night shifts (per month)**	
0	98 (31.0%)
≤ 5	66 (20.9%)
6–10	152 (48.1%)
**Department**	
Internal department	144 (45.6%)
Surgery department	77 (24.4%)
Gynecology and pediatrics	20 (6.3%)
ICU and ER	28 (8.9%)
Others	47 (14.9%)

### 4.2 Common method bias test

The Harman single-factor test was used to perform a factor analysis of the items involved in this study. The results indicated that there were 21 factors with eigenvalues greater than 1. The first factor explained 22.95% of the variance, which is below the threshold of 40% (Ding et al., 2025), indicating that there was no significant common method bias in this study.

### 4.3 Comparison of work engagement, occupational future time perspective, and career adaptability with different demographic characteristics

The results of the *t*-test and ANOVA showed significant differences in scores on several scales based on demographic characteristics and workplace factors. Preliminary analysis showed that married nurses had significantly higher work engagement levels than unmarried nurses, and supervisor nurses exhibited significantly higher work engagement and career adaptability than staff nurses and junior nurses. Nurses with formal employment had significantly higher adaptability than those with temporary contracts. Additionally, both work engagement and adaptability increased with longer tenure among newly recruited nurses. Nurses who did not work night shifts showed higher work engagement than those who worked ≤ 5 or 6–10 night shifts per month. The details are presented in Table 2.

**Table 2:** Differences in nurses' work engagement, OFTP, and career adaptability (*N* = 316).

**Characteristics**	**Work engagement**			**Occupational future time perspective**			**Career adaptability**		
	**M** ±**SD**	***t/F***	***P***	**M** ±**SD**	***t/F***	***P***	**M** ±**SD**	***t/F***	***P***
**Gender**									
Female	34.64 ± 10.58	0.672	0.502	28.60 ± 8.17	0.426	0.671	62.85 ± 14.08	−1.276	0.203
Male	32.62 ± 11.38			27.62 ± 7.95			68.54 ± 15.82		
**Education**									
Junior college	36.17 ± 11.64	0.144	0.886	28.58 ± 8.75	1.940	0.062	63.25 ± 14.31	0.674	0.510
Undergraduate	34.50 ± 10.63			27.48 ± 8.32			68.62 ± 16.11		
Postgraduate	34.46 ± 10.43			28.92 ± 7.33			67.98 ± 14.41		
**Marital status**									
Single	31.91 ± 6.45	-2.740	0.016	27.63 ± 7.98	-1.614	0.107	66.99 ± 15.54	-1.184	0.237
Married	37.00 ± 7.87			29.15 ± 8.23			69.15 ± 15.90		
**Title**									
Nurse	32.57 ± 10.64	2.717	0.018	27.60 ± 8.45	0.843	0.431	63.90 ± 15.71	3.874	0.022
Nurse practitioner	35.61 ± 10.92			28.41 ± 7.92			68.47 ± 16.23		
Supervisor nurse	38.47 ± 9.49			29.14 ± 8.20			70.32 ± 15.11		
**Employment types**									
Permanent	35.92 ± 10.86	1.446	0.340	31.42 ± 8.49	0.820	0.441	75.42 ± 10.82	1.270	0.282
Temporary	34.70 ± 9.56			29.51 ± 8.21			73.01 ± 10.02		
**Working years**									
< 1	33.32 ± 10.36	3.248	0.028	27.56 ± 6.15	0.962	0.383	64.70 ± 14.39	3.990	0.019
1–2	36.03 ± 10.51			28.89 ± 5.78			71.56 ± 15.67		
>2	38.66 ± 10.18			28.99 ± 5.31			69.05 ± 15.86		
**Night shifts (per month)**				
0	38.10 ± 9.94	2.803	0.042	28.54 ± 8.84	0.838	0.319	67.88 ± 11.92	0.346	0.792
≤ 5	34.14 ± 9.38			29.43 ± 7.33			69.36 ± 15.53		
6–10	35.83 ± 10.62			28.88 ± 7.68			68.43 ± 12.91		

### 4.4 Correlations between variables

The correlation analysis indicated that the work engagement of new nurses was positively correlated with career adaptability (*r* = 0.423, *P* < 0.01) and OFTP (*r* = 0.453, *P* < 0.01). In addition, the career adaptability of new nurses was positively correlated with OFTP (*r* = 0.410, *P* < 0.01). The results are shown in Table 3.

**Table 3:** Correlations among study variables.

**Variable**	**Work engagement**	**Career adaptability**	**Occupational future time perspective**
Work engagement	1	–	–
Career adaptability	0.423^**^	1	–
Occupational future time perspective	0.453^**^	0.410^**^	1

^**^ *P* < 0.01.

### 4.5 Multiple linear regression analysis of the factors affecting work engagement of new nurses

Using the total work engagement score of newly recruited nurses as the dependent variable, we entered as independent variables those characteristics that showed statistically significant differences in work-engagement comparisons (*P* < 0.05), together with the total scores of career adaptability and occupational future time perspective. Multivariable analysis indicated that marital status, professional title, length of service, number of night shifts, career adaptability, and occupational future time perspective were significant predictors of work engagement among newly recruited nurses (all *P* < 0.05), explaining 52.9% of the total variance (Table 4). Additionally, variance inflation factors (VIF) were all less than 5, indicating no multicollinearity among the variables.

**Table 4:** Multiple linear regression analysis of the factors affecting work engagement of new nurses.

**Variable**	***B***	**β**	**Beta**	***t***	***P***	**VIF**
Constant	13.385	3.261		4.105	< 0.001	1.621
Marital status	-1.263	1.314	-0.212	-2.200	0.041	1.405
Title	1.803	0.855	0.159	2.940	0.034	1.143
Working years	-1.268	0.746	-0.202	-2.359	0.042	1.674
Night shifts	1.211	0.614	0.183	2.343	0.020	1.792
Career adaptability	1.186	0.317	0.276	5.076	< 0.001	1.103
Occupational future time perspective	1.251	0.703	0.193	3.577	< 0.001	1.362

*R* = 0.727, *R*^2^ = 0.541, *AdjustedR*^2^ = 0.529; *F* = 81.579, *P* < 0.001.

### 4.6 Results of the structural equation model

**Figure 1:**
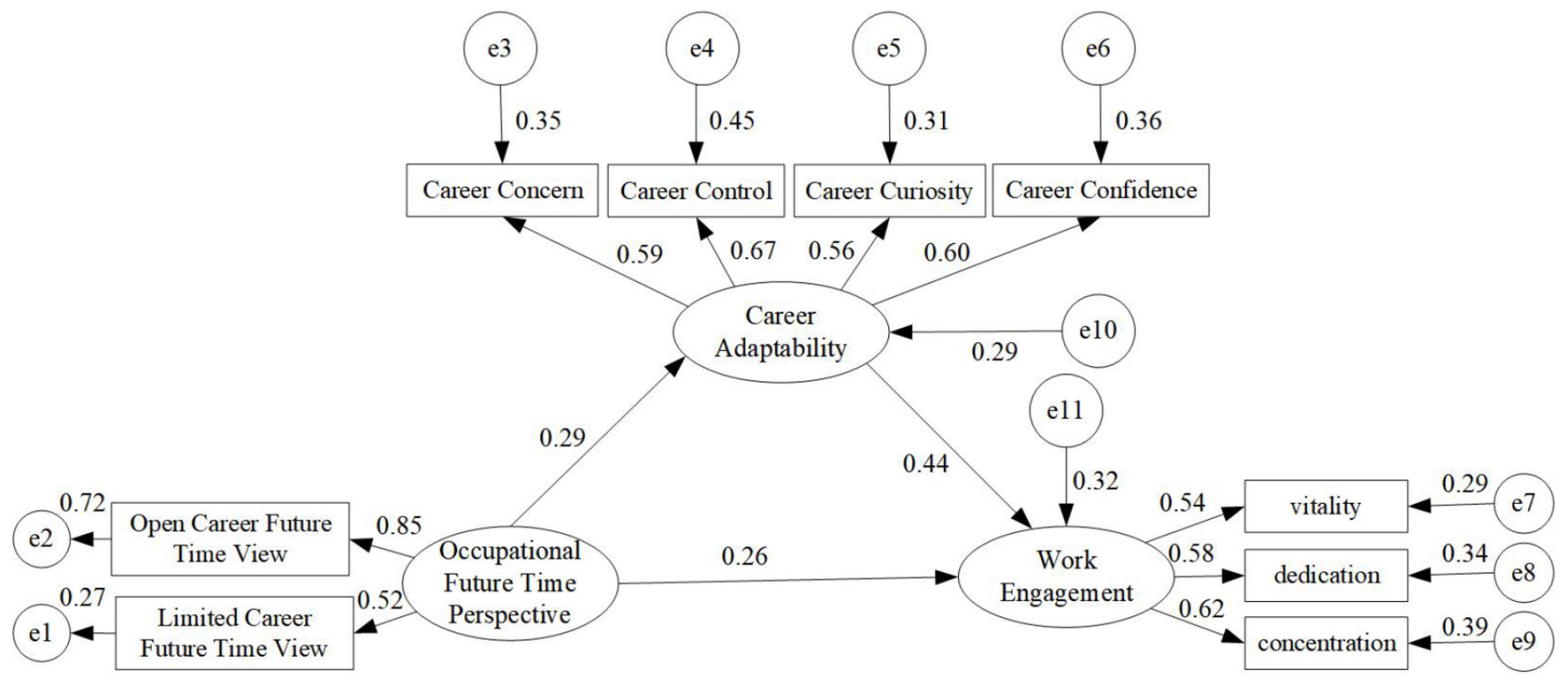
The results of SEM.

Structural equation modeling (SEM) was conducted to test the hypothesized model and the mediating role of career adaptability. The model fit indices indicated a good fit to the data [CMIN/DF = 1.486 (< 5), GFI = 0.976 (>0.90), AGFI = 0.955 (>0.90), CFI = 0.973 (>0.90), TLI = 0.959 (>0.90), RMSEA = 0.039 (< 0.05)]. The standardized path coefficients are presented in Figure 1. The OFTP of new nurses was positively correlated with career adaptability (β = 0.29, *P* < 0.01), and career adaptability was positively correlated with work engagement (β = 0.44, *P* < 0.01). Furthermore, the OFTP of new nurses directly affects work engagement (β = 0.26, *P* < 0.01). The 5,000 bootstrapping resamples revealed that the 95% confidence intervals (CI) for both mediation paths did not include 0, indicating significant mediation effects; the total indirect effect accounted for 44.61% of the total effect. Therefore, career adaptability acted as a mediator in the relationship between OFTP and work engagement (Table 5).

**Table 5:** Bootstrap test of mediation effect (normalized).

**Path**	**β**	**Boot SE**	**Bootstrap 95%CI**	***P***	**Percent (%)**
Total effect	0.295	0.051	0.120–0.468	< 0.001	–
Direct effect (OFTP → work engagement)	0.257	0.041	0.097–0.444	0.003	55.39
Indirect effect	0.129	0.034	0.055–0.240	< 0.001	44.61
OFTP → Career adaptability	0.295	0.016	0.120–0.438	0.001	14.83
Career adaptability → work engagement	0.438	0.087	0.271–0.602	< 0.001	29.78

## 5 Discussion

The present study aimed to explore the relationship between work engagement, occupational future time perspective, and career adaptability among new nurses in China. The results of our analysis provide valuable insights into how these factors interrelate and impact the overall work engagement of new nurses, a critical issue given the current nursing shortages and high turnover rates in the healthcare sector.

Our study highlights the significant impact of demographic and job-related characteristics on work engagement, OFTP, and career adaptability. For example, married nurses reported higher levels of work engagement compared to their unmarried counterparts. Furthermore, consistent with our bivariate analysis, nurses with formal employment contracts demonstrated significantly higher career adaptability than those with temporary contracts. It is important to note that in our enrolled cohort, women account for 92.1% and contract/temporary positions account for 83.2%, a distribution that accords with evidence on the female predominance of China's nursing workforce and the widespread use of contract-based employment in hospitals (Lu et al., 2021; Shang et al., 2014). Accordingly, the lower proportions of male and permanent-status nurses in our sample likely reflect real population features rather than sampling error. Nevertheless, we acknowledge that the small male and permanent-status subsamples constrain the precision of subgroup estimates. Future studies should therefore employ stratified sampling strategies to ensure these voices are adequately captured and to explore potential nuanced differences across these key demographics. Additionally, nurses with more years of experience and those who did not work night shifts or worked fewer night shifts per month also showed higher levels of engagement and adaptability. These findings suggest that personal and job-related factors play a crucial role in shaping new nurses' perceptions of their careers and their engagement at work. Nursing managers and educators should consider these factors when designing support programs and interventions for new nurses, paying attention to the life status of unmarried nurses, leveraging the experience of senior nurses, and building incentive mechanisms and support systems for night nurses to improve the work engagement of new nurses.

Our findings indicate that a positive OFTP significantly contributes to higher levels of work engagement among new nurses. This aligns with previous studies suggesting that individuals who have a clear and positive outlook on their future careers are more motivated and dedicated in their professional roles (Price and Reichert, 2017). Analyzing the reasons may be that a positive OFTP helps new nurses set long-term career goals and perceive a broader range of career opportunities, which are essential for navigating the initial challenges of their careers. These nurses tend to exhibit higher levels of vigor, dedication, and absorption in their work, key components of work engagement. This is particularly important in the nursing profession, where high levels of work engagement are associated with better patient care outcomes and lower turnover intentions (Moloney et al., 2018).

The role of career adaptability as a mediator between OFTP and work engagement is particularly noteworthy. The SEM analysis revealed that career adaptability significantly mediates the positive effect of OFTP on work engagement. Career adaptability comprises dimensions such as concern, control, curiosity, and confidence, which equip new nurses with the necessary skills and resources to manage career-related changes and challenges effectively (Koen et al., 2012). Our study found a significant positive correlation between OFTP and career adaptability, indicating that nurses who perceive more opportunities and a positive future in their career are more likely to develop strong adaptability skills. These skills, in turn, enhance their ability to remain engaged in their work despite the stresses and demands of the nursing profession.

This study extends Career Construction Theory and the Job Demands–Resources (JD–R) (Koekemoer et al., 2023) framework to explain how occupational future time perspective (OFTP) influences work engagement through career adaptability among new Chinese nurses. Rather than merely confirming prior findings, our results indicate that career adaptability functions as a key self-regulatory resource that transforms future-oriented motivation into sustained engagement. This suggests that interventions aimed at enhancing career adaptability could amplify the positive impacts of a positive OFTP on work engagement. For instance, training programs that focus on career planning, decision-making skills, and building confidence can empower new nurses, thereby increasing their engagement levels. These programs can help new nurses develop a proactive approach to their careers, making them more resilient and better prepared to handle the challenges they face in their early careers (Baharum et al., 2023). Additionally, the partial mediation observed (44.6%) suggests that career adaptability does not fully account for the relationship, implying that other personal or contextual factors (e.g., organizational support, leadership, and workload) may also contribute.

Within the Chinese nursing system, newly graduated nurses often encounter unstable employment contracts, heavy workloads, frequent night shifts, and limited decision-making authority. In this context, a strong occupational future time perspective may help sustain motivation despite uncertainty, while adaptability skills—planning, control, curiosity, and confidence—buffer stress, facilitate role transition, and promote retention. These mechanisms help explain why OFTP and adaptability were associated with higher engagement in our multi-site sample and align with national initiatives to build early-career resilience and capability.

From a managerial perspective, hospitals can enhance work engagement by embedding career-adaptability development into organizational practices. Practical steps include (i) establishing structured mentorship programs that foster career concern and confidence; (ii) incorporating adaptability training during induction with emphasis on goal setting, decision-making, and problem solving; (iii) providing transparent career pathways and opportunities for skill development; and (iv) moderating night-shift schedules and reducing contract insecurity to support well-being. These actions directly target the mechanisms identified here and translate theoretical insights into actionable workforce strategies.

In conclusion, this study contributes to a growing body of evidence that psychological resources—particularly future time perspective and adaptability—play a critical role in sustaining engagement during early nursing careers. By contextualizing these mechanisms within China's healthcare system, our findings enrich theoretical understanding and provide empirical guidance for interventions designed to strengthen resilience, retention, and professional growth among newly graduated nurses.

## 6 Limitations

This study has several limitations. First, the use of convenience sampling from 20 hospitals in specific provinces may limit the representativeness of the sample. Although the sample size was adequate, the geographic and institutional concentration may restrict the generalizability of the findings to new nurses in other regions or healthcare settings. Future studies should employ stratified or random sampling methods across more diverse regions and hospital types to enhance external validity. Additionally, as a cross-sectional study, our research may not account for causal relationships among occupational future time perspective, career adaptability, and work engagement. The underlying mechanisms and possible additional mediating effects require further investigation. Future studies could employ longitudinal designs to better capture the temporal dynamics and causal pathways between these variables. Third, the instruments used in this study relied on self-reported outcomes, which can introduce subjectivity into the results and potentially lead to bias in the collected data, thereby limiting the generalizability of the findings. Future research could incorporate multi-source data (e.g., organizational culture, leadership style) or objective indicators (e.g., workload records, performance metrics) to validate the findings. Additionally, qualitative interviews with multiple stakeholders, such as nursing staff, nurse managers, could validate the accuracy of the self-reported data.

## 7 Conclusions

In conclusion, this study underscores the importance of a positive occupational future time perspective in enhancing work engagement among new nurses. Secondly, it verifies the mediating effect of career adaptability between occupational future time view and work engagement. By fostering these attributes, nursing management can not only improve the immediate work experience of new nurses but also contribute to their long-term career development and retention. Addressing these aspects is vital for maintaining a motivated and engaged nursing workforce, ultimately leading to better patient care outcomes and addressing the ongoing challenges in the nursing profession.

## Data Availability

The raw data supporting the conclusions of this article will be made available by the authors, without undue reservation.
